# Case of Cyst Communicating with a Stomach Affected with Latent Cancer

**Published:** 1831

**Authors:** 


LXVIII.

### CASE OF CYST COMMUNICATING WITH A STOMACH AFFECTED WITH LATENT CANCER. By M. RIBES.

A MAN, aged 61 years, entered the infirmary on the 7th of August, 1829, complaining of pain in the epigastrium. His pulse was small, the tongue natural, the countenance pale. There was some œdema of the lower extremities. He had very little appetite, and the digestion was slow and painful. He had neither vomiting nor nausea—went regularly to stool, and the stethoscope shewed no disease of the lungs or heart. He was put upon soup and ptisans. On the 10th of August the patient felt in the line of the transverse arch of the colon some sharp pains, and his bowels were confined. Some aperient pills cleared the bowels. On the 13th he was again costive, and complaining of severe pains in the colon. He had a purgative lavement. 17th. The epigastric region was distended and sonorous. The stomach seemed to project, as it were, through the abdominal parietes. The lower part of the abdomen, from the umbilicus to the pubis, was soft and supple. A sulcus seemed to divide the abdomen transversely into two portions. The pain was always referred to the stomach and to the transverse arch of the colon. The left side of the chest appeared more dilated than the other, and the respiration was not now so clear on that side. The pulsations of the heart were obscure—the pulse slow—there was dyspnœa—and the appetite was almost annihilated.

Hitherto the complaint was considered to be chronic gastritis; but now the doctors became much puzzled, and they began to suspect that the patient laboured under tympanitis of the stomach complicated with pneumo-thorax of the left side. This uncertainty determined them to prescribe only for symptoms. Leeches were applied to the abdomen repeatedly, and six grains of calomel were given in six doses with two hours' interval. The patient was seized with a colliquative diarrhœa, and died on the 6th September.

*Dissection.* On opening the abdomen a membranous sac was observed lying transversely in the epigastric region. It was at first mistaken for the stomach; but they soon found that it was a cyst or pouch completely concealing that organ, to which, and to several other parts it was adherent. When opened, a gas was disengaged, and some sero-purulent liquid was found, holding in suspension some debris of false membranes, with which the whole of this bag was lined. There was a small hole communicating with the stomach. In the mucous membrane of the stomach was found a cancerous tumour partially ulcerated.—REVUE MEDICALE.

